# Quality of Life after Radical Prostatectomy: A Longitudinal Study

**DOI:** 10.3390/nursrep13030092

**Published:** 2023-08-08

**Authors:** Ana Anguas-Gracia, Isabel Antón-Solanas, Emmanuel Echániz-Serrano, Ana Belén Subirón-Valera, Beatriz Rodríguez-Roca, Raúl Juárez-Vela, Pedro José Satustegui-Dordá, María Teresa Fernández-Rodríguez, Vicente Gea-Caballero, Clara Isabel Tejada-Garrido, Ana Cobos-Rincón, Fernando Urcola-Pardo

**Affiliations:** 1 Department of Physiatry and Nursing, Faculty of Health Sciences, University of Zaragoza, 50009 Zaragoza, Spain; aanguas@unizar.es (A.A.-G.); ianton@unizar.es (I.A.-S.); eechaniz@unizar.es (E.E.-S.); subiron@unizar.es (A.B.S.-V.); brodriguez@unizar.es (B.R.-R.); pjsd@unizar.es (P.J.S.-D.); maitefer@unizar.es (M.T.F.-R.); furcola@unizar.es (F.U.-P.); 2 SAPIENF Research Group (B53_23R), University of Zaragoza, 50009 Zaragoza, Spain; 3 Research Group in Care (GIIS081), Institute for Health Research Aragón, University Clinical Hospital Lozano Blesa, 50009 Zaragoza, Spain; 4 Department of Nursing, Faculty of Health Sciences, University of La Rioja, 26004 Logroño, Spain; clara-isabel.tejada@unirioja.es (C.I.T.-G.); ana.cobos@unirioja.es (A.C.-R.); 5 GRUPAC, Research Group in Care, University of La Rioja, 26004 Logroño, Spain; 6 INCUisA Biomedical Research Center of La Rioja, 26004 Logroño, Spain; 7 Faculty of Health Sciences, Valencia International University, 46002 Valencia, Spain; 8 Community Health and Care Research Group, Faculty of Health Sciences, Valencian International University, 46002 Valencia, Spain

**Keywords:** quality of life, prostate, QLQ-C30, QLQ domain, urology

## Abstract

Background: Men with prostate cancer who undergo radical prostatectomy experience a decrease in quality of life, often related to sexual disfunction and urinary incontinence. Knowing and measuring the impact of radical prostatectomy on the individual’s social, emotional, and family quality of life could help to plan and develop an appropriate, patient-centred therapeutic approach. Aim: In this study, we aimed to evaluate changes in quality of life of patients with prostate cancer before and after radical prostatectomy. Methods: A longitudinal, observational study of 114 participants was conducted using the method of test–retest. Quality of life before and after radical prostatectomy was measured through the following self-administered questionnaires: (1) The EORTC QLQ-C30 in its Spanish version was used to assess the generic quality of life the participants; (2) the EORTC QLQ-PR25 in its Spanish version was used to assess the specific, health-related quality of life of prostate cancer patients. Results: A total of 114 men took part in this study. The results from the QLQ-C30 questionnaire indicated an improvement in the dimensions of emotional role and cognitive function, as well as in the symptoms of fatigue, pain, nausea and vomiting, insomnia, and loss of appetite, after surgery. Patients scored lower in the dimensions of role functioning, social function, and economic impact after radical prostatectomy. According to the results from the QLQ-PR25 questionnaire, 61.40% of the participants experienced sexual impotence and 26.31% suffered urinary incontinence after surgery. There were significant differences in some postsurgical outcomes between patients who had neurovascular bundles preserved and those who had not. Conclusions: In-depth knowledge of, and measurement of changes in, quality of life after radical prostatectomy should allow for comprehensive, multidisciplinary, patient-centred care planning. Psychosocial assessment, both before and after surgery, is crucial in patients with prostate cancer. This study was prospectively registered with the CEIC-A on 2012-06-27, with registration number C.P.-C.I. PI12/0088

## 1. Introduction

Quality of life (QOL) comprises several dimensions that are interrelated and that affect how people interact with their environment. Being ill affects not only people’s physical condition and well-being, but also their social and family roles [1,2]. People with cancer experience both functional and psychological changes that affect their daily lives, as well as their response to treatment and patient outcomes. 

Different types of cancer and cancer treatment have a different impact on patients. Therefore, it is important to take both elements into account when evaluating the quality of life of patients with cancer. Further, quality of life assessment tools must be able to discriminate between problems, measure the impact of treatment, and predict changes in quality of life in the long term. Test–retest studies measuring quality of life in patients with cancer are useful to describe such changes [3].

According to GLOBOCAN of the Global Cancer Observatory, prostate cancer is the most common cancer in men worldwide. In addition, it is the second cause of morbidity and fifth cause of mortality in men [4]. According to the latest estimates, the worldwide incidence of prostate cancer will increase to 1.7 million new cases and 499.000 deaths by 2030 [5]. In Spain, prostate cancer is the most frequent cancer in men and the fourth cause of mortality [6,7]. Early diagnosis through systematic screening of the population at risk, as well as adequate treatment choices, improves prognosis and increases survival rates [8].

Men who are newly diagnosed with prostate cancer can experience a significant level of anxiety and depression [9]. After radical prostatectomy, mental health issues are frequently associated with urinary and sexual symptoms, namely urinary incontinence, erectile dysfunction, and sexual impotence, resulting in symptoms of depression and reduced quality of life one year after surgery [10,11,12]. This can worsen men’s self-perception, mental health, social and family roles, as well as their coping mechanisms [13].

Previous investigations have suggested that men who have better disease-coping mechanisms are better positioned to successfully maintain their usual roles, carry out significant activities, find meaning in their lives, and maintain quality of life and physical, social, and emotional wellbeing. In contrast, men who struggle to adapt to their illness tend to lose hope and avoid committing to activities which, up to the point of diagnosis, used to be of vital importance to them [13,14].

Receiving a diagnosis of prostate cancer has serious repercussions on men’s social, emotional, and family lives. Thus, it is important to evaluate both disease-specific and generic quality of life before and after surgery to understand men’s perception of postsurgical changes and their ability to cope with them, as well as the symptoms of stress experienced, to plan adequate, patient-centred care interventions [15].

The aim of this investigation was to evaluate quality of life of men who underwent radical prostatectomy after diagnosis of prostate cancer both before and after surgery through two tools, namely the EORTC QLQ-C30 and the prostate cancer-specific EORTC QLQ-PR25. Both questionnaires are valid and reliable and have been used in previous studies with the same purpose [16].

## 2. Materials and Methods

### 2.1. Type of Study

A longitudinal, observational study of 114 patients was conducted, using a test–retest procedure, in which quality of life was assessed in patients undergoing radical prostatectomy through self-administered questionnaires. 

### 2.2. Data Collection

Patient recruitment took place from 1 January to 31 December 2019. Data collection was expected to finish on 31 August 2020, as post-test data were collected 8 months after radical prostatectomy. However, due to social restriction measures implemented by the Spanish government after the outbreak of the SARS-CoV-2 pandemic, 13 patients were lost to follow-up. Thus, in order to increase the sample, a second period of recruitment took place between 1 September 2020 and 31 May 2021. Data collection finished on 28 February 2022. Pre- and post-test measurements in the first period of data collection were 8 months apart. In the second period, they were 15 months apart due to the difficulties experienced during the pandemic. All the participants completed the questionnaires on two occasions: the first one (pre-intervention) on their admission before the surgery, while the second one (post-intervention) was at their second urological check-up, at least 8 months after the surgical discharge. 

All the participants were admitted to the urology department of the Miguel Servet University Hospital in Zaragoza, Spain. Inclusion criteria to take part in this study were (1) men aged 18 or over, (2) admitted to hospital for a planned radical prostatectomy, (3) with no cognitive impairment (assessed by the Pfeiffer test), (4) and who gave their informed consent to participate. All the patients who did not, or were unable to, complete either the pre-test, the post-test, or both were excluded from this investigation. 

This study was accredited by the European Organisation for Research and Treatment of Cancer (EORTC) for the use of the generic QOL questionnaire for cancer patients, EORTC QLQ-C30, and its specific module for prostate cancer patients, EORTC QLQ-PR25, using both validated versions into Spanish [17,18]. The QLQ-C30 questionnaire consists of 30 items measuring five functional scales (physical function, social function, emotional function, cognitive function, and role functioning), three symptom scales (fatigue, pain, nausea and vomit), a global quality of health scale and six single-item symptom scales assessing other cancer-related symptoms (dyspnoea, insomnia, appetite loss, constipation, diarrhoea, and economic impact) and its treatment. It is self-administered and its closed-ended questions refer to the patient’s situation or state during the last week. The first 28 items present Likert-type responses, with four possible options that show the degree of agreement or disagreement: 1-Not at all, 2-A little, 3-Somewhat, and 4-A lot. Items 29 and 30 are global scales with seven possible responses, ranging from 1-Very poor to 7-Excellent. All scores in this questionnaire are transformed into a scale from 0 to 100. Higher values on the global and functional health scales represent better QOL, while on the symptom scale they indicate a decrease in QOL due to cancer-associated symptoms [19,20].

The specific module for prostate cancer patients, the QLQ-PR25, is also self-administered and comprises 25 items measured on a 4-point Likert scale (1-Not at all, 2-A little, 3-Somewhat, and 4-A lot). It assesses the dimensions related to functional areas: urinary, bowel, and sexual symptoms; use of incontinence devices; and adverse effects derived from treatment, referring to their situation or state during the last week. Higher scores indicate a greater presence of symptomatology with worse outcomes in terms of health-related quality of life, except for three of the items in the sexual sphere where the interpretation is reversed, with higher scores indicating better sexual functioning [21,22,23]. Items 31 to 39 address urinary symptoms; items 40–43, 47 and 48 measure bowel symptoms; item 44 detects hot flushes. Quality of life in relation to sexual symptoms are measured by summing the score of items 49, 53–55 and subtracting the sum of items 50–52 from that number. Items 52–55 are valid only if there is sexual activity in the previous 4 weeks. 

### 2.3. Data Analysis

To simplify data analyses, some items were re-coded to homogenize the direction of the answers. Subsequently, we summed all the items in each scale and linearly transformed these scores into a scale ranging from 0 to 100. 

A descriptive study was carried out for all the variables included, with frequencies, percentages, means, and standard deviation, according to the type of variable. To analyse the changes that occurred in the dependent variable quality of life after the two measurements carried out, the mean and standard deviation of each of the dimensions of the questionnaires (QLQ-C30 and QLQ-PR25) were calculated, obtaining their frequency of change before (t1) and after surgery (t2). Parametric t-Student and ANOVA tests were used, with Tukey’s HSD test. A statistical significance of *p* < 0.05 at two-tailed was required for all contrast tests performed and a 95% confidence interval. SPSS V26. statistical software (IBM corporation, Chicago, IL, USA) for Windows, version 21.0, was used.

## 3. Results

### Description of the Study Population

A total of 114 men, aged 47 to 76, were included in this study. The mean age was 62.74 years (SD ± 6.73). Educational background was not reported by 65% of the participants; 13.2% had a university degree, 14% attended secondary school, and 15.8% attended primary school. The large majority of the patients were married (93%). All the patients but one were able to self-care. Similarly, all but one reported having a support network. In most cases, their spouse was identified as the main caregiver (92.1%). Regarding risk factors of prostate cancer, 18.4% had a family history of prostate cancer, 58.8% had smoked, and 72.7% had done so for over 2 decades. With regard to the surgical intervention, 57% were cases of open surgery. The neuromuscular bundles were maintained in 58.8% of the patients. As surgically derived sequelae, 61.4% of the patients presented sexual impotence and 26.3% urinary incontinence, both coexisting in 21% of the cases. All patients had a primary tumour type, whose stage was considered intermediate risk (Gleason = 7), with 7.9% having received previous treatment in the form of hormone therapy. (See Table 1).

We analysed patients’ generic quality of life before (t1) and after (t2) surgery. Patients experienced an improvement in their emotional role and cognitive function, as well as in the following symptoms: fatigue, pain, nausea and vomiting, insomnia, and appetite loss. In contrast, role functioning, social function, and economic impact worsened after surgery. 

The analysis of effect sizes found the greatest change in the dimensions of social function and economic impact (Table 2).

When comparing this change in the dimensions with the independent variables, the results show statistically significant differences in age, previous treatment, type of surgery performed, preservation or not of bundles, and smoking habits. Our findings suggest that symptoms of diarrhoea improved in patients aged 61–70 after surgery. Clinically significant changes were also observed in loss of appetite in patients from the age of 61 (effect size: 0.836) and in level of pain, which decreased in all age groups, especially in patients aged 61–70 (effect size: 0.697). 

We observed that role functioning significantly improved in patients who had received pre-surgical treatment. In contrast, social function worsened in both groups, although post-test scores were better in patients who had received pre-surgical treatment.

Significant changes were observed in emotional role and cognitive function, which improved more in patients who had undergone open surgery. 

Significant differences were found between patients who had neurovascular bundles preserved and those who had not. Thus, patients with preserved bundles scored higher in emotional role, insomnia, social, and cognitive function. With regard to health status, only those patients with preserved bundles improved their score in this dimension after surgery. 

Having a family history of prostate cancer improved patients’ cognitive function and pain, while not having smoked previously had a significant impact on appetite loss after radical prostatectomy (Table 3).

Analysis of the scores from the QLQ-PR25 before and after surgery suggest an improvement in intestinal symptoms and adverse effects. Urinary and sexual symptoms and use of incontinence devices worsened after surgery. Statistically significant differences pre- (t1) and post-surgery (t2) were found in all the dimensions but one, namely intestinal symptoms (Table 4). 

When grouped by age, sexual symptoms after surgery were worse in the group aged 61–70 (*p* = 0.019); patients aged 71 or over also experienced a worsening in sexual symptoms after surgery, but results were not statistically significant (*p* = 0.060) (Table 5).

These changes indicated a loss in terms of quality of life in relation to sexual symptoms, which also appeared when neurovascular bundles were preserved (*p* = 0.035) (Table 6). In the absence of pre-intervention treatment, a worsening of quality of life was observed in terms of incontinence device use (*p* = 0.037). No statistically significant associations were found between changes in the QLQ-PR25 quality of life dimensions and the other socio-demographic and clinical variables analysed.

These changes indicate that quality of life in relation to sexual symptoms worsened regardless of whether neurovascular bundles were preserved (*p* = 0.035) (Table 6). 

Significant differences before and after surgery were observed in the use of incontinence devices based on pre-surgical treatment (*p* = 0.037), with patients who had not received any treatment experiencing a worsening in the use of incontinence devices (Table 7). 

## 4. Discussion

This research has analysed the changes pre- and post-radical prostatectomy in both generic and prostate cancer specific quality of life in a single cohort of Spanish patients. The results have been analysed in the light of specific sociodemographic and clinical variables [24]. 

The sociodemographic characteristics of patients undergoing radical prostatectomy have changed in the last decade, with incidence increasing in patients aged 50–59. Patient age in our sample ranged from 47 to 76 years, with a mean age of 62.74. Similar findings were reported by Ji et al., who reported that over 10% of all new cases of prostate cancer in the USA were diagnosed in patients aged 55 or less [25]. Yet, other studies have reported a higher mean age of diagnosis [1,10,26]. This may be due to differences in the efficacy of screening and prevention campaigns, which allow for early diagnosis and improved patient outcomes.

Whilst pre- and post-radical prostatectomy assessment of patients’ generic (QLQ-C30) and specific (QLQ-PR25) quality of life allowed us to measure key dimensions and symptoms experienced by patients with prostate cancer, our investigation did not address each and every single factor affecting quality of life in this population, namely physical, mental, and social factors [27]. Cózar et al. agree with this argument and explain that prostate cancer exerts a significant influence on patients’ mental health [12]. Yet, few studies have examined the impact of mental health disorders on disease progression and patient outcomes in this population. 

We analysed patients’ generic quality of life before (t1) and after (t2) surgery through the QLQ-C [28]. Patients experienced an improvement in their emotional role and cognitive function, as well as in the symptoms of fatigue, pain, nausea and vomiting, insomnia, and appetite loss. In contrast, role functioning, social function, and economic impact worsened after surgery. Our findings coincide with those reported by Brassell et al. [28] However, other studies have reported an improvement in each and every single subscale and dimension after surgery in a similar sample [29]. 

The results from the QLQ-PR25 suggest that sexual and urinary symptoms, as well as use of incontinence devices, are the main causes of reduced quality of life after radical prostatectomy. Preservation of neurovascular bundles only improved intestinal symptoms and treatment adverse events after surgery.

Age was strongly linked to worsening of symptoms after surgery, and similar findings were reported by Holze et al. [30]. While their cross-sectional results suggested that younger men had better quality of life after surgery, their longitudinal analysis indicated that age was only associated with urinary incontinence. Our findings suggest that sexual symptoms and urinary incontinence affect patients’ perception of their quality of life, and that of their partners, and alter their physical, psychological, and social wellbeing. 

Two multivariate analyses described by de Nunzio and del Giudice determined that preoperative quality of life was an independent predictor of early incontinence and, also, that an independent association existed between metabolic syndrome alterations, adjuvant and salvage radiotherapy, and neurologic comorbidities, with a higher probability of needing surgery to treat urinary incontinence [31,32]. 

There are different treatment options for reducing sexual symptoms and recovering sexual potency. Whilst oral drugs, intracavernous injections, and penile implants may ameliorate these symptoms, it is important to evaluate their impact on quality of life from an integral approach [33]. It is also important to value non-pharmacological therapies capable of reversing erectile dysfunction with lasting and satisfactory results, used as a complement to pharmacological therapies in patients with organic, psychogenic, or mixed deterioration, or as a substitute in cases where drugs or surgery are not effective or not recommended [34]. In this way, it has been observed that constant and periodic exercises of the perineal muscles allow the improvement and recovery of normal erectile function in organic erectile dysfunction [35]. Specifically, as reported by Chung et al., previous studies have analysed the efficacy of these treatments from a predominantly sexual functional approach [36]. Yet, fewer investigations have analysed their impact on patients’ psychosocial health. In terms of the subjective impact of these treatments, it is important to consider that, whilst these treatments and devices can palliate post-surgical sexual sequels, they often affect physical, psychological, and social aspects of the life of an individual, causing problems including discomfort and altering men’s social role [37,38]. Psychosocial interventions and patient-centred care in patients with prostate cancer improves wellbeing to some extent, although no statistically significant improvement has been demonstrated in disease-specific symptoms, self-efficacy, uncertainty, anxiety, and depression [39]. On the other hand, post-surgical changes in quality of life based on age affect patients’ psycho-emotional sphere and are associated with risk of depression and associated comorbidity, including low self-esteem and feelings of anguish, anxiety, guilt, and uncertainty.

## 5. Limitations 

The public safety measures implemented in Spain, derived from the SARS-CoV-2 pandemic, delayed data collection. To avoid possible bias in the assessment of quality of life, data pertaining to participants diagnosed with COVID-19 between the two rounds of questionnaires were withdrawn from this investigation.

Our participants were recruited using a convenience sampling technique in a single, large Spanish hospital, which may limit the representativeness of the sample. We also wish to acknowledge the fact that this investigation has focused primarily on physical sequalae. Yet, psychological and psychosocial sequalae following radical prostatectomy can be severe and should be addressed in future research in this area.

## 6. Conclusions

The generic questionnaire QLQ-C30 demonstrated a global positive change in quality of life after radical prostatectomy, and a significant negative change in aspects related to social function and economic impact. The specific questionnaire QLQ-PR25 identified a loss in quality of life in relation to increased use of urinary incontinence products and sexual disfunction. Sexual symptoms were worse in patients aged 61–70, independently of preservation of neurovascular bundles. 

These results suggest improving areas for the improvement of the nurses’ approach to the sexual function and the QOL of patients undergoing a prostatectomy. An early assessment of the different QOL areas would allow the development of rational interventions of the nurses responsible of the health education of these patients and their main carers. This training should include specific interventions from the preoperative period to the definitive discharge in order to teach the correct use of incontinency products and also the correct time and exercises to develop a good physical rehabilitation. 

As we recommend this assessment, it is important to highlight that these tools can offer an unbalanced view of quality of life in this group of patients, as they focus primarily on physical sequalae. Knowing and measuring the impact of these changes on the patient’s social, affective, and personal quality of life will allow health practitioners to plan and develop adequate therapeutical interventions. Future research on this topic should assess mental disorders and psychological symptoms using specific questionnaires.

## Figures and Tables

**Table 1:** Sociodemographic and clinical variables.

		Total (N = 114)	Freq (%)
Age (years)	≤60	39	34.2%
	61–70	60	52.6%
	≥71	15	13.2%
Education	Primary	18	15.8%
	Secondary	16	14%
	University	15	13.2%
	Unknown	65	57%
Marital status	Married	106	93%
	Single	5	4.4%
	Widower	3	2.6%
Family support network	Yes	113	99.1%
	No	1	0.9%
Primary support person	Wife	104	92.1%
	Women with sons	3	2.6%
	Sons	2	1.8%
	Brothers	4	3.5%
Self—care capacity	Yes	113	99.1%
	No	1	0.9%
Profession	Retired	64	56%
	Self-employed	19	17%
	Employed	28	24.4%
	Not included	3	2.6%
Family history of prostate cancer	Yes	21	18.4%
	No	93	81.6%
Smoking	No	47	41.2%
	Yes	67	58.8%
Type of approach performed in the surgery	Open	65	57%
	Laparoscopic	49	43%
Preservation of neurovascular bundles	Yes	67	58.8%
	No	47	41.2%
Presence of sexual impotence after surgery	Yes	70	61.4%
	No	44	38.6%
Presence of urinary incontinence after surgery	Yes	30	26.3%
	No	84	73.7%
Preoperative treatment	Yes	9	7.9%
	No	105	92.1%
Total		114	100%

Pre- and post-test assessment of patients’ quality of life using the generic cancer questionnaire EORTC QLQ-C30.

**Table 2:** Change in the dimensions of the QLQ-C30 for the overall sample.

QLQ-C30 Dimension	Before	After	Change (CI. 95%)	*t* ^1^ (Significance)	Effect Size
Overall health status	73.54 (13.59)	70.76 (15.17)	2.78	1.856	0.205
			(−0.19; 5.74)	(0.066)	
Physical function	93.57	94.33	−0.76	−0.829	0.086
	(8.87)	(8.33)	(−2.58; 1.06)	(0.409)	
Role functioning	93.27	86.99	6.29	3.099	0.439
	(14.32)	(18.12)	(2.27; 10.30)	(0.002)	
Emotional role	77.27	86.77	−9.50	−5.959	0.640
	(14.84)	(11.93)	(−12.66; −6.34)	(<0.001)	
Cognitive function	93.13	99.56	−6.43	−5.906	0.550
	(11.69)	(2.68)	(−8.59; −4.27)	(<0.001)	
Social function	94.15	73.68	20.47	10.752	1.608
	(12.73)	(19.94)	(16.70; 24.24)	(<0.001)	
Fatigue	8.19	4.00	4.19	3.864	0.372
	(11.26)	(8.64)	(2.04; 6.34)	(<0.001)	
Pain	10.23	6.58	3.65	2.078	0.244
	(14.96)	(13.78)	(0.17; 7.14)	(0.040)	
Nausea and vomit	1.46	0.00	1.46	2.748	0.257
	(5.68)	(0.00)	(0.41; 2.52)	(0.007)	
Dyspnoea	5.56	3.22	2.34	1.421	0.160
	(14.65)	(10.83)	(−0.92; 5.60)	(0.158)	
Insomnia	23.98	11.70	12.28	5.375	0.471
	(26.05)	(19.32)	(7.75; 16.81)	(<0.001)	
Appetite loss	3.51	0.58	2.92	2.985	0.262
	(11.19)	(4.40)	(0.98; 4.86)	(0.003)	
Constipation	3.51	4.39	−0.88	−0.653	0.086
	(10.27)	(11.32)	(−3.54; 1.78)	(0.515)	
Diarrhoea	3.51	0.00	3.51	3.646	0.342
	(10.27)	(0.00)	(1.60; 5.42)	(<0.001)	
Economic impact	2.34	29.24	−26.90	−10.665	2.339
	(11.50)	(26.29)	(−31.90; −21.90)	(<0.001)	

Note = mean (standard deviation). ^1^ Related-samples *t*-test.

**Table 3:** Statistically significant changes in QLQ-C30 measurements based on independent variables.

QLQ-C30 Dimension	Before	After	Change (CI. 95%)	*t* (Significance)	Effect Size
Constipation Age 61–70	3.23 (9.94)	0.00 (0.00)	−3.23 (9.94)	0.032	0.483
Role functioning No pre-surgical treatment	94.29 (13.64)	86.35 (18.46)	−7.94 (21.32)	−2.991 (0.003)	1.357
Pre-surgical treatment	81.48 (17.57)	94.44 (11.79)	12.96 (16.20)		
Cognitive function No pre-surgical treatment	92.54 (12.01)	99.52 (2.79)	6.98 (11.96)	−1.965 (0.049)	0.825
Pre-surgical treatment	100.0 (0.00)	100.0 (0.00)	0.00 (0.00)		
Social function No pre-surgical treatment	94.29 (12.62)	72.86 (19.92)	−21.43 (20.64)	−1.991 (0.047)	0.823
Pre-surgical treatment	92.59 (14.70)	83.33 (18.63)	−9.26 (12.11)		
Emotional role Open surgery	74.74 (14.58)	87.18 (11.51)	12.44 (17.87)	2.152 (0.034)	0.436
Laparoscopy	80.61 (14.67)	86.22 (12.56)	5.61 (15.16)		
Cognitive function Open surgery	91.03 (12.18)	99.49 (2.90)	8.46 (12.19)	2.181 (0.031)	0.441
Laparoscopy	95.92 (10.50)	99.66 (2.38)	3.74 (10.36)		
Overall health status Preserved bundles	75.35 (13.79)	78.55 (10.09)	3.20 (15.30)	3.504 (0.001)	0.604
Non-preserved bundles	72.26 (13.40)	65.30 (15.80)	−6.97 (15.19)		
Emotional role Preserved bundles	77.66 (14.55)	93.62 (8.00)	15.96 (17.27)	3.561 (0.001)	0.614
Non-preserved bundles	76.99 (15.15)	81.97 (11.93)	4.98 (15.42)		
Cognitive function Preserved bundles	90.78 (12.44)	100.0 (0.00)	9.22 (12.44)	2.121 (0.037)	0.375
Non-preserved bundles	94.78 (10.94)	99.25 (3.47)	4.48 (10.69)		
Social function Preserved bundles	96.81 (8.95)	81.56 (15.63)	−15.25 (15.48)	2.495 (0.014)	0.404
Non-preserved bundles	92.29 (14.60)	68.16 (20.87)	−24.13 (22.53)		
Insomnia Preserved bundles	22.70 (27.89)	2.13 (8.24)	−20.57 (28.28)	−2.962 (0.004)	0.544
Non-preserved bundles	24.88 (24.85)	18.41 (21.93)	−6.47 (19.45)		
Cognitive function Previous family history	88.89 (13.26)	99.21 (3.64)	10.32 (13.41)	−2.002 (0.045)	0.247
No previous family history	94.09 (11.17)	99.64 (2.43)	5.55 (11.08)		
Pain Previous family history	7.94 (16.35)	9.52 (11.27)	1.59 (16.59)	−2.242 (0.025)	0.205
No previous family history	10.75 (14.67)	5.91 (14.25)	−4.84 (19.13)		
Appetite loss Non-smokers	4.26 (11.24)	0.00 (0.00)	−4.26 (11.24)	0.033	0.536

Pre- and post-test assessment of patients’ quality of life using the specific prostate cancer questionnaire EORTC QLQ-PR25.

**Table 4:** Change in the dimensions of the QLQ-PR25 for the overall sample.

Dimension QLQ-PR25	Before	After	Change (CI. 95%)	*t* ^1^ (Significance)	Effect Size
Urinary scope	11.57	12.80	−1.23	−2.898	0.336
	(2.32)	(4.62)	(−2.07; −0.39)	(0.005)	
Intestinal symptoms	6.43	6.25	0.18	1.572	0.224
	(0.61)	(0.96)	(−0.05; 0.40)	(0.119)	
Sexual symptoms *	−3.34	1.11	−4.45	−12.18	1.393
	(2.46)	(3.79)	(−5.17; −3.72)	(<0.001)	
Use of incontinence devices	0.02	2.76	−2.75	−26.34	5.165
	(0.14)	(0.74)	(−2.95; −2.54)	(<0.001)	
Adverse effects of treatment	3.39	3.05	0.34	4.406	0.583
	(0.78)	(0.26)	(0.19; 0.50)	(<0.001)	

* Only patients/subjects with sexual activity within the last four weeks. Mean (standard deviation). ^1^ Related-samples *t*-test.

**Table 5:** Changes in the dimensions of the QLQ-PR25 according to patient age.

QLQ-PR25 Dimension	Before	After	Change (CI. 95%)	Significance	Effect Size
Urinary scope ≤60	11.36 (2.24)	11.79 (4.24)	0.43 (4.15)	0.404	0.268
61–70	11.65 (2.38)	13.27 (4.75)	1.62 (4.60)	0.663	0.282
≥71	11.85 (2.38)	13.54 (4.94)	1.69 (5.25)	0.999	0.013
Intestinal symptoms≤60	6.38 (0.59)	6.36 (1.14)	−0.02 (1.37)	0.568	0.191
61–70	6.50 (0.65)	6.23 (0.93)	−0.27 (1.18)	0.940	0.098
≥71	6.23 (0.44)	6.08 (0.28)	−0.15 (0.55)	0.942	0.109
Sexual symptoms ≤60	−4.21 (2.08)	1.00 (3.83)	5.21 (3.99)	0.650	0.179
61–70	−3.16 (2.68)	1.35 (3.92)	4.51 (3.78)	0.019	0.876
≥71	−1.62 (1.04)	0.23 (3.06)	1.85 (3.29)	0.060	0.721
Use of incontinence devices≤60	0.00 (0.00)	2.62 (0.77)	2.62 (0.77)	0.743	0.236
61–70	0.03 (0.18)	2.83 (0.75)	2.80 (0.76)	0.917	0.172
≥71	0.00 (0.00)	2.75 (0.71)	2.75 (0.71)	0.985	0.066
Adverse effects ≤60	3.28 (0.72)	3.00 (0.00)	−0.28 (0.72)	0.699	0.173
61–70	3.48 (0.82)	3.06 (0.25)	−0.42 (0.86)	0.880	0.164
≥71	3.31 (0.75)	3.15 (0.55)	−0.16 (0.99)	0.549	0.306

**Table 6:** Evolution in the dimensions of the QLQ-PR25 according to the preservation of bundles.

QLQ-PR25 Dimension	Before	After	Change (CI. 95%)	*t*-Test (Significance)	Effect Size
Urinary scope Preserved	11.30 (2.23)	11.70 (4.32)	0.40 (4.42)	−1.640 (0.104)	0.314
Not preserved	11.76 (2.38)	13.57 (4.70)	1.81 (4.54)		
Symptoms intestinal Preserved	6.43 (0.62)	6.06 (0.25)	−0.37 (0.70)	−1.404 (0.163)	0.270
Not preserved	6.43 (0.61)	6.39 (1.22)	−0.04 (1.43)		
Sexual symptoms Preserved	−4.62 (2.41)	0.74 (4.07)	5.36 (13.43)	2.129 (0.035)	0.404
Not preserved	−2.45 (2.09)	1.36 (3.59)	3.81 (3.68)		
Use of incontinence devices Preserved	0.00 (0.00)	2.73 (0.88)	2.73 (0.88)	−0.072 (0.943)	0.026
Not preserved	0.03 (0.17)	2.78 (0.68)	2.75 (0.69)		
Adverse effects Preserved	3.19 (0.61)	3.00 (0.00)	−0.19 (0.61)	1.759 (0.081)	0.317
Not preserved	3.54 (0.86)	3.09 (0.34)	−0.45 (0.94)		

**Table 7:** Evolution in the dimensions of the QLQ-PR25 according to pre-surgical treatment.

Dimension QLQ-PR25	Before	After	Change (CI. 95%)	Z Test (Significance)	Size of the Effect
Urinary symptoms No	11.65 (2.36)	12.86 (4.74)	1.21 (4.69)	−0.659 (0.510)	0.051
Yes	10.67 (1.66)	12.11 (3.02)	1.44 (1.74)		
Intestinal symptoms No	6.44 (0.62)	6.28 (1.00)	−0.16 (1.23)	−0.286 (0.775)	0.143
Yes	6.33 (0.50)	6.00 (0.00)	−0.33 (0.50)		
Sexual symptoms No	−3.42 (2.51)	1.13 (3.79)	4.55 (3.88)	−1.220 (0.223)	0.341
Yes	−2.44 (1.59)	0.78 (3.96)	3.22 (4.15)		
Use of incontinence devices No	0.02 (0.15)	2.84 (0.74)	2.82 (0.75)	−2.088 (0.037)	0.889
Yes	0.00 (0.00)	2.17 (0.41)	2.17 (0.41)		
Adverse effects No	3.40 (0.79)	3.06 (0.27)	−0.34 (0.84)	−0.041 (0.967)	0.012
Yes	3.33 (0.71)	3.00 (0.00)	−0.33 (0.71)		

No other significant associations were found in specific quality of life measured by the QLQ-PR25 in relation to the rest of the sociodemographic and clinical variables.

## Data Availability

Data Availability will be under request to the first author.

## Public Involvement Statement

No public involvement in any aspect of this research.

## Guidelines and Standards Statement

This manuscript was drafted against the Strengthening the Reporting of Observational Studies in Epidemiology (STROBE) Statement (von Elm, E., Altman, D.G., Egger, M. et al. The Strengthening the Reporting of Observational Studies in Epidemiology (STROBE) statement: guidelines for reporting observational studies [published correction appears in *Ann. Intern Med.* 15 January 2008; 148(2): 168]. *Ann. Intern Med.* 2007; 147(8): 573–577. doi:10.7326/0003-4819-147-8-200710160-00010) observational research.
